# Locating tandem repeats in weighted sequences in proteins

**DOI:** 10.1186/1471-2105-14-S8-S2

**Published:** 2013-05-09

**Authors:** Hui Zhang, Qing Guo, Costas S Iliopoulos

**Affiliations:** 1 College of Computer Science and Technology, Zhejiang University of Technology, Hangzhou, Zhejiang 310023, China; 2 Corresponding author. College of Computer Science and Engineering, Zhejiang University, Hangzhou, Zhejiang 310027, China; 3 Department of Computer Science, King's College London Strand, London WC2R 2LS, England

## Abstract

A weighted biological sequence is a string in which a set of characters may appear at each position with respective probabilities of occurrence. We attempt to locate all the tandem repeats in a weighted sequence. A repeated substring is called a tandem repeat if each occurrence of the substring is directly adjacent to each other. By introducing the idea of equivalence classes in weighted sequences, we identify the tandem repeats of every possible length using an iterative partitioning technique. We also present the algorithm for recording the tandem repeats, and prove that the problem can be solved in *O*(*n*^2^) time.

## Introduction

A weighted biological sequence, called for short a *weighted sequence*, is a special string that allows a set of characters to occur at each position of the sequence with respective probability, instead of a fixed single character occurring in a normal string. It can be viewed as a compressed version of *multiple alignment *which shows strength in extracting and representing the conserved commonalities of a set of sequences.

Weighted sequences are apt at summarizing poorly defined short sequences, e.g. transcription factor binding sites, the profiles of protein families and complete chromosome sequences[1]. With this model, one can attempt to locate the motifs of biological importance, to estimate the binding energy of the proteins, even to infer the evolutionary homology. It thus exhibits theoretical and practical significance to design powerful algorithms on weighted sequences in proteins.

This paper concentrates on locating those tandem repeats in a weighted sequence. Tandem repeats occur in a string when a substring is repeated for two or more times and each repetition is directly adjacent to each other. For example, The substring *ATT *occurs in the string *X = CATT ATT ATTG *for three times, and each occurrence of *ATT *is consecutive, one after the other. Then *ATT *is a tandem repeat of length 3 of *X*.

The motivation for investigating tandem repeats in weighted sequences comes from the striking feature of DNA that vast quantities of tandemly repetitive segments occur in the genome, with high proportion of more than 50 percent in fact[2]. Some examples are microsatellite, minisatellite, and satellite DNA.

It should be noticed that tandem repeats are not redundant information, but of either functional or evolutionary significance [3]. For instance, tandem repeats frequently occur within or in the proximity of genes, i.e., either in the untranslated regions up and downstream of open reading frames, within introns, or in coding regions[4]. Recent evidence supports that tandem repeats in these regions can play a significant role in regulating gene expression and modulating gene function[5]. Thus it is of great biological interest to locate tandem repeats in biological DNA sequences and proteins.

It has been an effort for a long time to identify special areas in a biological sequence by their structure. Large amount of work has been done to find all tandem repeats in non-weighted strings. Technically, these solutions can be divided into two main categories. One employed traditional string comparison and searching method, where the most famous algorithms were Crochemore's partioning[6] and LZ decomposition[7], with time complexity *O*(*n *log *n*) respectively. The other computed tandem repeats by constructing suffix tree and suffix array. Although needing extra memory, these algorithms can also reach *O*(*n *log *n*) time by limiting the number of output[8-12].

However, relatively less work has been studied in weighted sequences circumstance. Iliopoulos et al.[13,14] were the first to touch this field, and extract repeats and other types of repetitive motifs in weighted sequences by constructing weighted suffix tree. Weighted suffix tree was built simulating suffix tree, with the distinction that the weight of each substring should be considered. This directly led to a big size and its strong dependence on the presence probability of the weighed suffix tree. Another solution[15,16] used the partitioning technique based on KMR algorithm to find tandem repeats of length *d *in *O*(*n *log *d*) time. But they did not give efficient algorithm for computing the tandem repeats of all lengths.

On the other hand, a lot of recent results of studies on identifying hot spots in proteins enlightened us. Huang et al.[17] firstly utilized the support vector machine(SVM) classifier based upon the hydropathy blocks to classify protein sequences. Then Xia et al.[18] used support vector machine (SVM) to predict hot spot residues in protein interfaces. Selecting nine individual features from 62 features, they developed a new ensemble classifier APIS to further improve the prediction accuracy. You et al.[19] developed a robust manifold embedding technique for assessing the reliability of interactions and predicting new interactions, which was reinterpreted into the problem of measuring similarity between points of its metric space after transforming a given PPI network into a low dimensional metric space using manifold embedding based on isometric feature mapping. Zheng et al.[20] employed independent component analysis for gene selection, then introduced gene selection and explicitly enforcing sparseness into nonnegative matrix factorization for tumor clustering. Wang et al.[21] proposed a novel tumor classification method based on correlation filters other than the model to identify the overall pattern of tumor subtype hidden in genes.

The paper focuses on finding tandem repeats of all length in a given weighted sequence in proteins. The paper is organized as follows. In the next section we give the necessary theoretical preliminaries used, then introduce the all-tandem-repeats problem and explains why Crochemore's partitioning algorithm cannot be adapted to weighted sequences. After that, we present our algorithm for computing all the tandem repeats in weighted sequences, and give experimental results to verify the algorithm's performance. Finally we conclude and discuss our research interest.

## Preliminaries

A biological sequence used throughout the paper is a string either over the 4-character DNA alphabet *Σ *={A,C,G,T} of nucleotides or the 20-character alphabet of amino acids. Assume that readers have essential knowledge of the basic concepts of strings, now we extend parts of them to weighted sequences. Formally speaking:

**Definition 1 **Let an alphabet be *Σ *= {*σ*_1_, *σ*_2_, . . . , *σ*_l_}. A weighted sequence *X *over *Σ*, denoted by *X *[1, *n*] = *X*[1]*X *[2] . . . *X*[*n*], is a sequence of *n *sets *X*[*i*] for 1 ≤ *i *≤ *n*, such that:

$$X\left[i\right]=\left\{\left(\sigma_{j},\pi_{i}\left(\sigma_{j}\right)\right)|1\;\leq j\leq l,\;\pi_{i}\left(\sigma_{j}\right)\;\geq 0,and\;\sum_{j=1}^{l}\pi_{i}\left(\sigma_{j}\right)=1\right\}$$

Each *X*[*i*] is a set of couples (*σ_j_*, *π_i _*(*σ_j_*)), where *π_i_*(*σ_j_*) is the non-negative *weight *of *σ_j _*at position *i*, representing the probability of having character *σ_j _*at position *i *of *X*.

Let *X *be a weighted sequence of length *n*, *σ *be a character in *Σ*. We say that *σ occurs *at position *i *of *X *if and only if *π_i_*(*σ*) > 0, written as *σ *∈ *X*[*i*]. A nonempty non-weighted string *f*[1,*m*] (*m *∈ [1, *n*]) *occurs *at position *i *of *X *if and only if position *i *+ *j *− 1 is an occurrence of the character *f *[*j*] in *X*, for all 1 ≤ *j *≤ *m*. Then *f *is said to be a *factor *of *X*, and *i *is an *occurrence *of *f *in *X*.

The probability of the presence of *f *at position *i *of *X *is called the *weight *of *f *at *i*, written as *π_i_*(*f*), which can be obtained by using different weight measures. We exploit the one in common use, called the *cumulative weight*, defined as the product of the weight of the character at every position of $f:\pi_{i}\left(f\right)=\;\prod_{j=1}^{m}{\pi_{i}{}_{+j}}_{-1}\left(f\left[j\right]\right).$

Considering the following weighted sequence of length 5:

(1) $$X=\left\{\begin{array}{c} \left(A,0.5\right)\\ \left(C,0.25\right)\\ \left(G,0.25\right) \end{array}\right\}G\left\{\begin{array}{c} \left(A,0.6\right)\\ \left(C,0.4\right) \end{array}\right\}\left\{\begin{array}{c} \left(A,0.25\right)\\ \left(C,0.25\right)\\ \left(G,0.25\right)\\ \left(T,0.25\right) \end{array}\right\}C$$

the weight of *f *= *GAT *at position 2 of *X *is: *π*_2_(*f*) = 1 × 0.6 × 0.25 = 0.15. That is, *GAT *occurs at position 2 of *X *with probability 0.15. Note that for clarity, we employ a simplified vertical representation method for a weighted sequence, where the probability 1 can be ignored by simply remaining the character with probability 1.

A factor *f *of a weighted sequence *X *is called a *repeat *in *X *if there exist at least two distinct positions of *X *that are occurrences of *f *in *X*. As a special case of repeat, tandem repeats can be formally defined as follows.

**Definition 2 **A factor *f *of length *p *of a weighted sequence *X *is called a *tandem repeat *in *X *if there exists a triple (*i*, *f*, *l*) such that for each 0 ≤ *j *<*l *− 1, position *i *+ *jp *is an occurrence of the factor *f *in *X*.

It is easy to see that, the difficulty for locating the tandem repeats in weighted sequences arises from uncertainties of weighted sequences. Firstly, different characters might occur at the same position, which yields multiple factors of equal length at each position of the weighted sequence. Secondly, as each character occurs at one position with respective probability, the corresponding factors produced also have different presence probabilities, thus the weight of each appearance of a factor *f *can be highly different.

As scientists pay more attention to the pieces with high probabilities in DNA sequences, we fix a constant threshold for the presence probability of the motif, that is, only those occurrences with probability not less than this threshold are counted.

**Definition 3 **Let *f *be a factor of length *d *of a weighted sequence *X *that occurs at position *i*, a real constant threshold *k *≥ 1. We say that *f *is a real factor of *X *if and only if the weight (probability) of *f *at *i*, *π_i_*(*f*), is at least $\frac{1}{k}$. Exactly, $\prod_{j=1}^{d}{\pi_{i}{}_{+j}}_{-1}\left(f\left[j\right]\right)\geq \frac{1}{k}.$

In the above example (1), set 1/*k *= 0.3, then *AGA *is a real factor of *X *that occurs at position 1 since *π*_1_(*AGA*) = 0.5 × 1 × 0.6 = 0.3 ≥ 0.3, while *CAC *is not a real factor of *X *at position 3 since *π*_3_(*CAC*) = 0.1 < 0.3.

## The all-tandem-repeats problem

Now we introduce the all-tandem-repeats problem in weighted sequences as below:

**Problem 1 **Given a weighted sequence *X*[1, *n*] and a real constant *k *≥ 1, the *all-tandem-repeats *problem identifies the set *S *of all triples (*i*, *f*, *l*), where 1 ≤ | *f *| ≤ *n*/2 and *f *is a real factor of *X*.

Our algorithm for picking all the tandem repeats is based on the following idea of equivalence relation on positions of a string:

**Definition 4 **Given a string *x *of length *n *over *Σ*, an integer *p *∈{1, 2, . . . , *n*}, *S *be a set of positions of *x*: {1, 2, . . . , *n *− *p *+ 1}, then *E_p _*is defined to be an equivalence relation on *S *such that: for two positions *i*, *j *∈ *S*, (*i*, *j*) ∈ *E_p _*if *x*[*i*, *i *+ *p *− 1] = *x*[*j*, *j *+ *p *− 1].

In the following context, a nonempty substring of *x *of length *p *is called a *p-substring *of *x*. Clearly, two positions *i *and *j *of *x *are said to be *p*-equivalent when two *p*-substrings starting at *i *and *j *in *x *are identical. Although this definition is defined on non-weighted strings, it can also be extended to weighted sequences. Before presenting our algorithm, we first introduce Crochemore's partitioning algorithm[6] for computing tandem repeats in non-weighted sequences. The algorithm employs the following idea of equivalence class and partition.

**Definition 5 **Consider the substring *w *= *x*[*i*, *i *+ *p *− 1] for *i *∈ *S*. The set of all positions of *x *that are related to *i*, i.e, {*j*|(*i*, *j*) ∈ *E_p_*, *j *∈ *S*}, is called the *equivalence class *of *i*, or alternatively, the equivalence class associated with *w*, denoted by *C_w_*.

**Definition 6 **Let *S*_1_, *S*_2_, . . . , *S_r _*be nonempty subsets of *S*, we say that {*S*_1_, *S*_2_, . . . , *S_r_*} is a *partition *of *S *if:

(i) *S *= *S*_1 _∪ *S*_2 _∪ . . . ∪ *S_r_*

(ii) *S_i_*∩ *S_j_*= Ø for 1 ≤ *i*, *j *≤ *r *and *i *≠ *j*.

For an equivalence relation *E_p _*on a set *S*, all the equivalence classes of *E_p_*, called *E_p_*-*classes*, compose a partition of *S*, since every element of *S *falls into exactly one *E_p_*-class. We also say that *S *is partitioned into a family of *E_p_*-classes. In this sense, partitions and equivalence relations are the same.

It is obvious that each *E_p_*-class of cardinality not less than two records the occurrences of a repetitive *p*-substring of *x*. Hence, the problem of computing all the repeated *p*-substrings of *x *can be rephrased as finding the partition of *E_p_*.

Observe that *E_p_*_+1 _is a refinement of *E_p _*by excluding the position *n *− *p *+ 1. Thus the equivalence relations can be iteratively constructed by starting with *E*_1_, then successively building *E*_2_, *E*_3_, etc., until *E_L _*such that each *E_L_*-class is a singleton who refers to a set that consists of only one element. Crochemore efficiently executed this iterative computation and located all the tandem repeats in *x *in *O*(*nlogn*) time by introducing the following ideas:

- *Small-classes: *Consider the refinement from *E_p _*to *E_p_*_+1 _. Assume that an *E_p_*-class *C *is partitioned into *r E_p_*_+1_-classes, we call the one of maximal size a *big class *of *C*, and the other *r *− 1's are *small classes*.

- *Smaller-half trick : *The trick depends on the following Lemma:

**Lemma 1 ***Let × be a string of length n*, *p *∈ {1, 2, . . . , *n*}, *i*, *j *∈ {1, 2, ... , *n *− *p*}. *Then:*

$$\left(i,\;j\right)\in E_{p+1}\Leftrightarrow \left(i,\;j\right)\in E_{p}\text{ and }\left(i+\text{1,}\;\;j\;+\;1\right)\in \text{a small }E_{p}\text{ - class}$$

Therefore, instead of partitioning all *E_p_*-classes at stage *p*, the algorithm simply examines each small *E_p_*-class *SC *and partitions those related classes *RC *such that {*RC*| *i *∈ *RC *and *i *+ 1 ∈ *SC*}. Simply speaking, for any *E_p_*-class *C*, only the positions that will be transferred into small *E_p_*_+1_-classes are assigned new indexes, while the big *E_p_*_+1_-class directly inherits the index of *C*.

The running time of this algorithm is proportional to the union of small classes. By definition, all the *E*_1_-classes are small, with cardinality less than *n*. As each small *E_p_*_+1_-class has the size not greater than half of the cardinality of its corresponding *E_p_*-class, a position cannot belong to a small class more than *logn *times. Therefore, the partitioning algorithm takes *O*(*nlogn*) time for a string of length *n*.

Although proved to be optimal, this algorithm cannot conform to a weighted sequence *X *due to the following reasons:

1. Multiple distinct characters may occur at the same one position of a weighted sequence. In this case, a position may goes to more than one equivalence classes associated with different substrings of the same length, thus the smaller-half trick makes no sense.

2. In weighted sequence circumstance, the presence probability of any factor should not be ignored as it is restricted by the probability threshold.

## Our algorithm

As we stated above, Crochemore's algorithm cannot be directly used in weighted sequence, but it enlightens us to borrow the idea of partitioning. By improving the method for computing repeated patterns in weighted sequences we proposed in [22], we first simulate the definition for *E_p_*-classes of non-weighted strings, and give the corresponding weighted version:

**Definition 7 **Consider a factor *f *of length *p *in a weighted sequence *X*[1, *n*]. An *E_p_*-class associated with *f *is the set *C_f_*(*p*) of all position-probability pairs, denoted by (*i*, *π_i_*(*f*)), such that *f *occurs at position *i *with probability *π_i_*(*f*) ≥ 1/*k*.

*C_f_*(*p*) is an ordered list that contains all the positions of *X *where *f *occurs. Note that only the occurrences of those real factors are considered. For this reason, the probability of each appearance of a factor should be recorded and kept for the next iteration.

Although tandem repeats are special cases of repeats in weighted sequences, the following facts draw a distinction between the algorithms for computing tandem repeats and the repeats we proposed before.

**Fact 1 ***The occurrences of a tandem repeat are not overlapping*.

**Fact 2 ***If a factor f is a tandem repeat of X, any consecutive alignment of f should not be reported as a tandem repeat again*.

For instance, a string *AT AT AT AT *will report a tandem repeat (1, *AT*, 4), not (1, *AT AT*, 2). According to the above facts, tandem repeats can be timely filtered during the construction of equivalence classes.

Note that in this construction process, a position *i *is allowed to go to several but no more than |*Σ*| different *E_p_*-classes, due to the uncertainty of weighted sequences. Though, we follow to use the notion "partition" to describe the process of building *E_p_*-classes from *E_p_*_−1_-classes, which can be computed based upon the following corollary:

**Corollary 1 ***Let p *∈ {1, 2, . . . , *n*}, *i*, *j *∈ {1, 2, ... , *n *− *p*}. *Then:*

((*i*, *π_i_*(*f*)), (*j*, *π_j_*(*f*))) ∈ *C_f_*(*p*) *iff *((*i*, *π_i_*(*f*'), $\left(j,\;\pi_{j}\;\left(f^{\prime }\right)\right)\in C_{f^{\prime }}\left(p\;-1\right)$*and *((*i *+ *p *− 1, *π_i_*_+_*_p_*_−1_(*σ*)), (*j *+ *p *− 1, *π_j_*_+_*_p_*_−1_(*σ*))) ∈ *C_σ_*(1)

*where σ *∈ *Σ*, *f and f*'*are two factors of length p and p *− 1 *respectively, such that f *= *f*'*σ and π_i_*(*f*) ≥ 1/*k*, *π_j_*(*f*) ≥ 1/*k*.

Our algorithm for picking all the tandem repeats of *X *then operates as follows:

1. "Partition" all the *n *positions of *X *to build *E*_1 _and detect all the tandem repeats of length 1: For every character *σ *∈ *Σ*, create a class *C_σ_*(1) that is an ordered list of couples (*i*, *π_i_*(*σ*)), where *i *is an occurrence of *σ *in *X *with probability not less than 1/*k*. Each class composed of more than one element forms *E*_1_. Those *C_σ_*(1)s in which the distance between two or more adjacent position *i *is 1 report the tandem repeats of length 1.

2. Iteratively compute *E_p_*-classes from *E_p_*_−1_-classes using the above corollary for *p *≥ 2, and find all the tandem repeats of length *p*: Take each class *C*(*p *− 1) of *E_p_*_−1_, partition *C*(*p *− 1) so that any two positions *i*, *j *∈ *C*(*p *− 1) go to the same *E_p_*-class if positions *i *+ *p *− 1, *j *+ *p *− 1 belongs to a same *E*_1_-class, and this *E_p_*-class represents a real factor of *X*.

3. For each *E_p_*-class *C*(*p*) partitioned by *C*(*p *− 1), test if the factor associated with *C*(*p*) is a tandem repeat of *X*: If the cardinality of *C*(*p*) is at least two and any distance between two or more adjacent positions in *C*(*p*) equals *p*, add the corresponding triple into the tandem repeat set  S. Eliminate those *C*(*p*)s who are singletons, and keep the rest to proceed the iterative computation at stage *p *+ 1.

4. The computation stops at stage *L*, once no new *E_L_*_+1_-classes can be created or each *E_L_*-class is a singleton.

**Algorithm 1 **Compute all the tandem repeats of a weighted sequence

**Input: **a weighted sequence *X*[1, *n*], *k *≥ 2 ∈ *R*

**Output: **all the tandem repeats of *X*

1:    **Algorithm **Compute-Tandem-Repeats(*X*, *k*)

2:    **for ***i *← 1 **to ***n ***do**

3:    *l *← 0

4:    **for ***j *← 1 **to **|*Σ*| **do**

5:    **for **each *σ_j_*∈ *X*[*i*] **do**

6:    **while **${\pi_{i}}_{+l}\left(\sigma_{j}\right)\;\geq \frac{1}{k}$**do**

7:    add(*i *+ *l*, *π_i_*+*l*(σ_j_)) to $C_{\sigma_{j}}$(1)

8:    *l *← *l *+1

9:    **if ***l *> 1 **then**

10:    $S\leftarrow S\cup \left(i,\sigma_{j},l\right)$

11:    *p *← 1

12:    **while **$p\leq \frac{n}{2}$**and **there is a non-singleton class *C*(*p *− 1) of *E_p_*_−1 _or *E_p_*_−1_≠ Ø **do**

13:    (*C_f_*(*p *− 1), *f*) ← extract a pair from *E_p_*_−1 _list

14:    SUB ← Create-Equiv-Class(*C_f_*(*p *− 1), *f*)

15:    *p *← *p *+ 1

16:    add SUB to *E_p_*

We use a doubly linked list to store each equivalence class, which needs *O*(*n*) space for a bounded-size alphabet. The computation for tandem repeats is demonstrated as Algorithm 1, which repeatedly calls function Create-Equiv-Class. Algorithm 2 depicts the procedure to construct all possible *E_p_*-classes from a certain *E_p_*_−1_-class, and report those tandem repeats of length *p*. It is easy to see that Algorithm 1 takes *O*(*n*^2^) time for a constant-size alphabet, since each refinement of *E_p _*from *E_p_*_−1 _costs linear time, and there are *O*(*n*) stages in total. The running time of Algorithm 2 is proportional to the size of the given *E_p_*_−1_-class, since tandem repeats of length *p *are reported along with the partitioning of the given *E_p−_*_1_-class. Taking all the *E_p_*_−1_-classes into account, stage *p *requires *O*(*n*) time and *O*(*n*) extra space. Thus the overall time complexity of finding all tandem repeats of every possible length amounts to *O*(*n*^2^).

**Algorithm 2 **Identify tandem repeats of length *p*

**Input: **An *E_p_*_−1_-pair: class *C_f_*(*p *− 1), a factor *f *corresponding to *C*_*p*−1_

Output: All the E*_p_*-pairs derived from the input

1:    **Function **Create-Equiv-Class(*C_f _*(*p *− 1), *f*)

2:    **for **each (*i*, *π_i _*(*f*)) ∈ *C_f_*(*p *− 1) **do**

3:        *l *← 0

4:        **for **each *σ_j_*∈ *X *[*i *+ *p *− 1] **do**

5:            *f_j_*← *fσ_j_*

6:            *π_i _*(*f_j_*) ← *π_i _*(*f*) × *π_i _*+ *p *− 1(*σ_j_*)

7:            **while **$\pi_{\text{i}+l}\left({\text{f}}_{j}\right)\geq \frac{1}{k}$**do**

8:                add(*i *+ *l*, *π_i_*+*l*(*j*)) to $C_{f_{j}}$(*p*)

9:                *l *← *l *+1

10:          **if ***l *> 1 **then**

11:              $S\leftarrow S\cup \left(i,f_{j},l\right)$

12:    **for **each *j ***do**

13:          **if **|$C_{f_{j}}$ (*p*)| = 1 **then**

14:              delete $C_{f_{j}}$ (*p*)

15:          **else**

16:              add $\left(C_{f_{j}}\left(p\right),f_{j}\right)\text{ to}\;E_{p}$

17:    **return ***E_p_*

**Theorem 1 ***The all-tandem-repeats problem can be solved in O(n*^2^*) time*.

## Experimental results

To verify the running time of our algorithm, we implemented the algorithm, programmed in C++, for locating all the tandem repeats in a given weighted sequence. The experiment environment is a Intel Core2 Duo CPU P8700 2.5GHz system, with 2GB of RAM, under the Microsoft Windows XP operating system (SP2).

**Figure 1:**
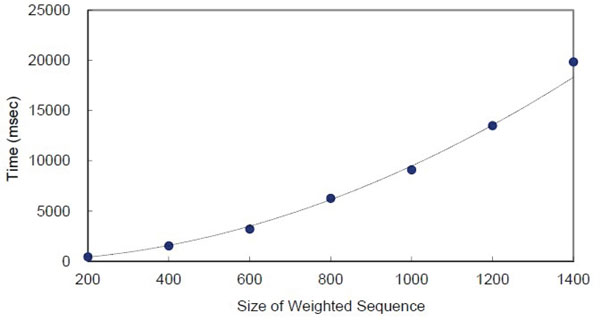
**Time consumption with respect to *n***.

In our experiments, the family of SR (serine/arginine rich) proteins SC35 across species and alleles was used. We transformed the alignment of the sequences [23] to a weighted sequence as the input data. Firstly, we fixed the presence probability threshold to be a small constant, then simply tested the performance of the algorithm with respect to the size of the weighted sequence, denoted by *n*. In this case, set the constant 1/*k *= 0.01. Figure 1 demonstrates the running time curve of our algorithm with respect to *n*. It is easily observed that, the algorithm runs in *O*(*n*^2^) time as expected.

**Figure 2:**
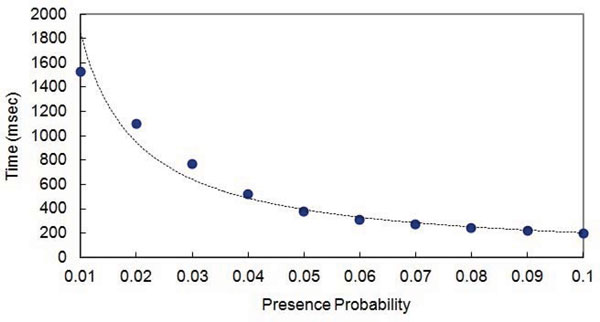
Time consumption with respect to the threshold 1 / *k*.

As we stated before, our algorithms is heavily dependent on the presence probability. We then fixed the size of the input weighted sequence to be 400, and executed our algorithm considering different presence probabilities. Figure 2 gives the time consumption of the the algorithm with respect to the presence probability 1/*k*. Clearly, the running time grows exponentially as the probability threshold gets smaller.

## Conclusions

The paper investigated the tandem repeats arisen in weighted sequences. As opposed to the non-weighted version, the uncertainty of weighted sequences and the presence probability of every character in the sequence must be considered. We devised efficient algorithm for identify all the tandem repeats in a weighted sequence, which operates in *O*(*n*^2^) time.

Note that if |*Σ*| are sufficiently large, the total number of repeats might be very huge. In the worst case, i.e. each character of *Σ *appears at every position of the weighted sequence, the total number of repeats of a weighted sequence can be exponential, that is *O*(|*Σ*|*^n^*). This fact of considering equivalence-classes of positions seems to lead to a quadratic algorithm. If |*Σ*| is relatively small, and the number of weighted positions in the weighted sequence is bounded, the algorithm appears to be running in *O*(*n*^2^) time as expected.

## Competing interests

The authors declare that they have no competing interests.

## Declarations

The authors declare that funding for publication of the article was sponsored by National Key Technology R&D Program.

This article has been published as part of *BMC Bioinformatics *Volume 14 Supplement 8, 2013: Proceedings of the 2012 International Conference on Intelligent Computing (ICIC 2012). The full contents of the supplement are available online at http://www.biomedcentral.com/bmcbioinformatics/supplements/14/S8.
